# Supplementary material for: Baseline Characterization of the Gut Microbiota of Field and Colony Populations of Phlebotomus tobbi and Preliminary Assessment of the Anti-Leishmanial Activity of Cultivable Bacteria
Source: Pathogens. 2026 Jun 23;15(7):658. doi: 10.3390/pathogens15070658 (PMC13416292; doi:10.3390/pathogens15070658)
Supplement: Supplementary file 1 [file pathogens-15-00658-s001.zip › pathogens-4357168-File S1/Taxa2ASV_Decomposer/Family/f__Xanthomonadaceae/heatmap_with_clustering.pdf]

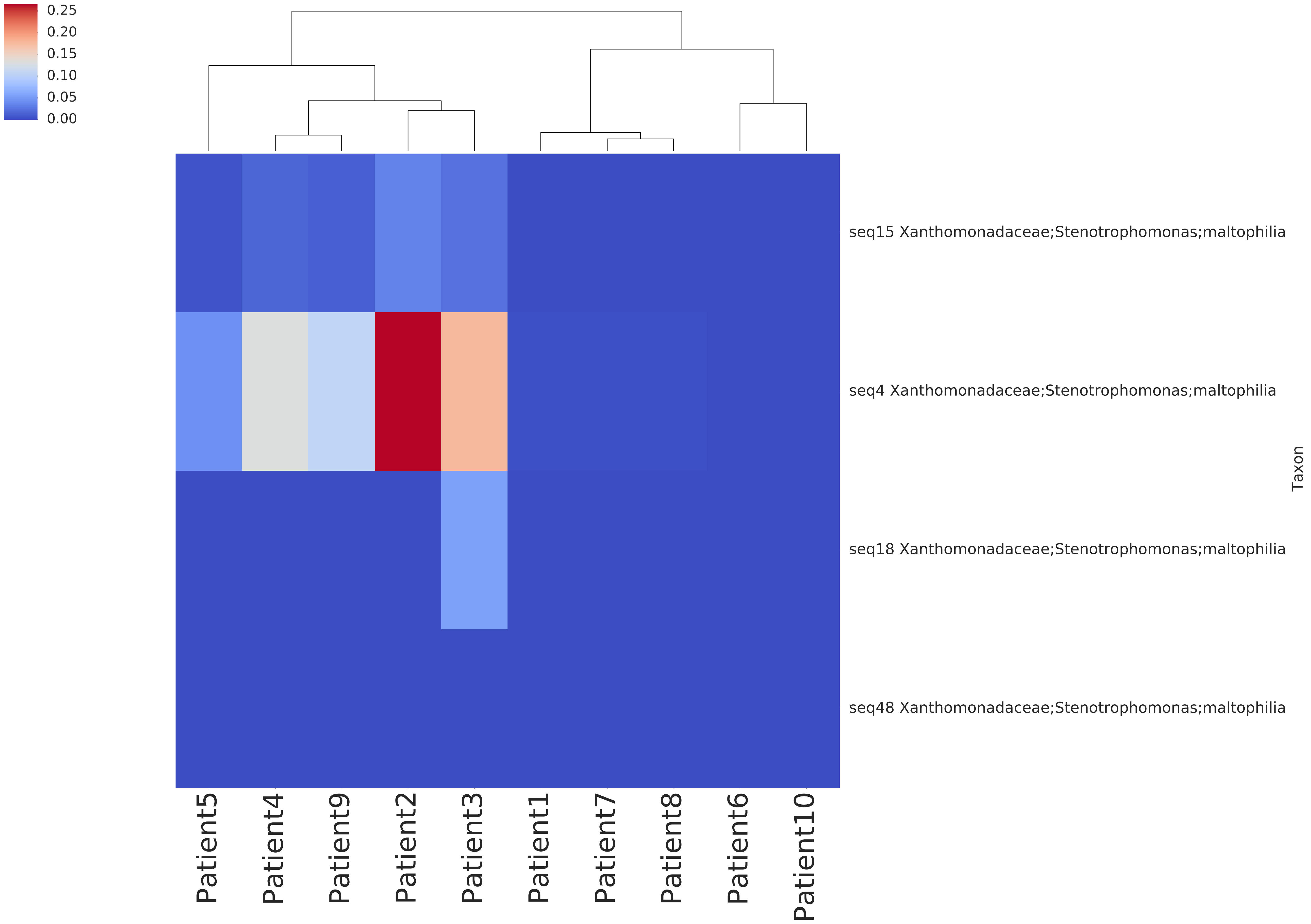

0.25
0.20
0.15
0.10
0.05
0.00
seq15 Xanthomonadaceae;Stenotrophomonas;maltophilia
seq4 Xanthomonadaceae;Stenotrophomonas;maltophilia
seq18 Xanthomonadaceae;Stenotrophomonas;maltophilia
seq48 Xanthomonadaceae;Stenotrophomonas;maltophilia
Taxon
Patient5
Patient4
Patient9
Patient2
Patient3
Patient1
Patient7
Patient8
Patient6
Patient10
